# Financial Incentives for Increasing Uptake of HPV Vaccinations: A Randomized Controlled Trial

**DOI:** 10.1037/hea0000088

**Published:** 2014-08-18

**Authors:** Eleni Mantzari, Florian Vogt, Theresa M. Marteau

**Affiliations:** 1 Centre for the Study of Incentives in Health and Department of Psychology, King’s College London; 2 Institute of Pharmaceutical Science, King’s College London; 3 Centre for the Study of Incentives in Health and Department of Psychology, King’s College London and Behaviour and Health Research Unit, University of Cambridge

**Keywords:** financial incentives, vouchers, HPV vaccination, human papillomavirus

## Abstract

***Objective:*** Uptake of human papillomavirus (HPV) vaccinations by 17- to 18-year-old girls in England is below (<35%) target (80%). This trial assesses (a) the impact of financial incentives on uptake and completion of an HPV vaccination program, and (b) whether impacts are moderated by participants’ deprivation level. It also assesses the impact of incentives on decision quality to get vaccinated, as measured by attitudes toward the vaccination and knowledge of its consequences. ***Method:*** One thousand 16- to 18-year-old girls were invited to participate in an HPV vaccination program: 500 previously uninvited, and 500 unresponsive to previous invitations. Girls randomly received either a standard invitation letter or a letter including the offer of vouchers worth £45 (€56; $73) for undergoing 3 vaccinations. Girls attending their first vaccination appointment completed a questionnaire assessing decision quality to be vaccinated. Outcomes were uptake of the first and third vaccinations and decision quality. ***Results:*** The intervention increased uptake of the first (first-time invitees: 28.4% vs. 19.6%, odds ratio [*OR*] = 1.63, 95% confidence interval [CI; 1.08, 2.47]; previous nonattenders: 23.6% vs. 10.4%, *OR* = 2.65, 95% CI [1.61, 4.38]) and third (first-time invitees: 22.4% vs. 12%, *OR* = 2.15, 95% CI [1.32, 3.50]; previous nonattenders: 12.4% vs. 3%, *OR* = 4.28, 95% CI [1.92, 9.55]) vaccinations. Impacts were not moderated by deprivation level. Decision quality was unaffected by the intervention. ***Conclusions:*** Although the intervention increased completion of HPV vaccinations, uptake remained lower than the national target, which, in addition to cost effectiveness and acceptability issues, necessitates consideration of other ways of achieving it.

Human papillomavirus (HPV) is a ubiquitous sexually transmitted virus that could lead to cervical cancer (Brabin et al., 2005; Moscicki et al., 1998), the second most common cancer in women worldwide (Ferlay et al., 2010). HPV vaccines help prevent infection with “high-risk” strands of HPV that are associated with later cancer development (Ault, 2007; Franco & Harper, 2005). Immunization against HPV requires completion of three vaccinations to effectively reduce the risk of cervical cancer (Ault, 2007; Joura et al., 2007). The degree of protection afforded by incomplete immunization is currently unknown (Widdice, Bernstein, Leonard, Marsolo, & Kahn, 2011).

Since September 2008, a national program has been implemented in England and Wales, United Kingdom, aiming to vaccinate girls aged 12 to 13 years against HPV. A 2-year “catch-up” campaign targeting 17- to 18-year-old girls has also been initiated. The objective of these vaccination programs is to provide three doses of the HPV vaccine to females before they become sexually active, when the risk of HPV infection and subsequent cervical cancer development increases. It is estimated that if this objective is met and vaccination coverage is sufficiently high (80% of the target population), up to 400 deaths per year in England could be prevented (Sheridan, White, Barlow, & Soldan, 2010). Although the national program targeting 12- to 13-year-olds has met the 80% uptake target set by the National Health Service (NHS),^1^ with 88.1% of girls receiving the first vaccination, and 80.1% receiving the third, the catch-up campaign targeting 17- to 18-year-olds has resulted in below-target uptake, with 62.2% of girls receiving the first vaccination and only 31.8% receiving the third (Sheridan et al., 2010). Apart from these cohort differences, uptake of the HPV vaccine in England is also marked by social inequalities, with girls living in deprived areas and from ethnic minority backgrounds being less likely to get vaccinated (Roberts et al., 2011). Women from these populations are also more likely to develop cervical cancer (Shack, Jordan, Thomson, Mak, & Møller, 2008).

Offering girls financial incentives to undergo the HPV vaccination could increase uptake rates. Financial incentives are increasingly being considered and used in health care policies in the United Kingdom and elsewhere in an attempt to improve health (Lagarde, Haines, & Palmer, 2007; Le Grand, 2008). They are most effective in promoting “one-off” behaviors, such as getting vaccinated (Achat, Mcintyre, & Burgess, 1999; Seal et al., 2003; Sutherland, Christianson, & Leatherman, 2008). Indeed, findings from systematic reviews suggest that financial incentives can increase uptake of recommended vaccinations by both adults and children (Briss et al., 2000; Giuffrida & Torgerson, 1997; Kane, Johnson, Town, & Butler, 2004; Ndiaye et al., 2005; Stone et al., 2002; Sutherland et al., 2008). Their effectiveness is predicted to depend on recipients’ level of social and material deprivation. Consequently, one potential advantage of using financial incentives to promote health is that they may be more effective in motivating behavior change in the most socially deprived (Sutherland et al., 2008). When applied to HPV-vaccination programs, incentives therefore have the potential not only to improve the below-target uptake rates but also to reduce social inequalities. Most of the calls to use incentives in HPV-vaccination programs in the United Kingdom, however, have focused on incentivizing vaccination providers (e.g., general practitioners) rather than vaccination recipients (Tanday, 2008). The effectiveness of financial incentives in this latter context is therefore currently unknown. Furthermore, no studies have assessed the role of social deprivation in the moderation of the impact of financial incentives on vaccination uptake.

The exact mechanisms by which financial incentives operate to influence behavior, including vaccination uptake, are currently unknown. In theory, they are likely to work via learning-theory principles, by linking the target behavior to a positively evaluated stimulus (e.g., a reward with monetary value), thus strengthening the value associated with it (Marteau, 2010). From an economics perspective, this increases the utility gained from performing the target behavior, thus providing an impetus for individuals to act (Camerer & Hogarth, 1999; Dawes, 1999; Hertwig & Ortmann, 2001; Lopes, 1994; Zwick, Erev, & Budescu, 1999). At the same time, financial incentives might work by shifting people’s expectations of the likely consequences of the target behavior in a positive direction, or by facilitating allocation of limited cognitive capacity, in such a way as to achieve the now more highly valued target behavior (Marteau, 2010). These possibilities suggest that financial incentives may enable people to overcome the costs and barriers associated with initiating the target behavior and/or shift their perception of the related cost–benefit ratio, such that the benefits of performing the related behavior outweigh the costs. Certain theories, such as motivation crowding theory (Frey & Jegen, 2001) and cognitive evaluation theory (Deci & Ryan, 1985), predict that the offer of incentives might sometimes have the opposite effect to the one intended. However, the extent to which this can occur in the context of health-related behaviors has been questioned (Promberger & Marteau, 2013).

Even if effective in increasing uptake of HPV vaccinations, the use of financial incentives in HPV-vaccination programs will need to be considered in the context of their possible negative consequences. Unlike most interventions designed to change behavior, the use of financial incentives raises particular concerns regarding their potentially adverse effects on the quality of people’s decisions to engage in incentivized behaviors. For example, it has been argued that the prospect of receiving a financial reward could result in the risks associated with the incentivized behavior being overlooked (Marteau, Ashcroft, & Oliver, 2009). This is particularly relevant to behaviors associated with physical and/or psychological side effects, such as getting vaccinated. To date, no known studies have assessed the impact of financial incentives on the quality of decisions to engage in incentivized behaviors.

Assessing the quality of health-related decisions is important, as it allows predictions to be made regarding the psychological and physiological adjustment of patients (Johnston & Vögele, 1993; Marteau, Dormandy, & Michie, 2001). One way to judge the quality of a decision to engage in a behavior is to assess whether it represents an informed choice. An informed choice has been operationally defined as one that is based on knowledge of the relevant information, is consistent with the decision-maker’s values, and is behaviorally implemented (Marteau et al., 2001). These three dimensions of informed choice are also echoed in definitions of informed decision making (Bekker, Hewison, & Thornton, 2004; Dowie, 2002; Irwig, McCaffery, Salkeld, & Bossuyt, 2006; Jepson, Hewison, Thompson, & Weller, 2005; Rimer, Briss, Zeller, Chan, & Woolf, 2004) and clinical decision quality (Sepucha, Fowler, & Mulley, 2004). Using this operationalization a multidimensional measure of informed choice has been developed (Marteau et al., 2001), validated (Michie, Dormandy, & Marteau, 2002), and used to assess the quality of decisions in the context of screening (e.g., Dormandy, Michie, Hooper, & Marteau, 2006; Dormandy, Tsui, & Marteau, 2007; Jaques, Sheffield, & Halliday, 2005; Kellar et al., 2008; Mathieu et al., 2007; Michie, Dormandy, & Marteau, 2003; Smith et al., 2010). Given that both screening and vaccination uptake involve preventative behaviors with potential side effects, this measure can readily be used to assess decision quality in the context of HPV vaccination uptake.

The present study is the first trial that addresses the aforementioned uncertainties with regard to the use of financial incentives in HPV vaccination programs. The specific aims of this trial are (a) to assess the impact of financial incentives on initial uptake and completion of an HPV-vaccination program, and (b) to examine whether the impact of financial incentives on uptake and completion of an HPV-vaccination program is moderated by recipients’ level of social deprivation. The trial further aims to assess the impact of financial incentives on the quality of decisions to be vaccinated, as measured by participants’ attitudes toward the vaccination and their knowledge of its health consequences. It was hypothesized that girls who were offered financial incentives to get vaccinated against HPV would be more likely to receive the first and third HPV vaccinations. Participants’ level of social deprivation was hypothesized to moderate the effects of financial incentives on uptake of the first and third HPV vaccinations, with larger effects of the incentives being predicted for the most socially deprived. In line with existing concerns, it was further hypothesized that compared with nonincentivized girls, those offered financial incentives would have less positive attitudes toward the HPV vaccination and would be less knowledgeable about its health consequences.

## Method

### Trial Design

The present study is is a parallel-group randomized controlled trial. Further details of the study methods are available in the trial protocol (Mantzari, Vogt, & Marteau, 2012b).^2^

### Context

Between 2008 and 2009, the uptake rates in England for the “catch-up” HPV-vaccination campaign, targeting females aged 17 to 18 years, was 62.2% for the first vaccination, 54.2% for the second vaccination, and 31.8% for the third vaccination (Sheridan et al., 2010). The equivalent figures for the Birmingham East and North (BEN) Primary Care Trust, where this trial was conducted, were 72.2%, 64.6%, and 34.2%, respectively (Sheridan et al., 2010).

### Participants

Participants were one thousand 16- to 18-year-old girls: 500 had not yet received an invitation to attend the vaccination program (first-time invitees), and 500 had previously received an invitation to get vaccinated, but had failed to attend the first vaccination appointment (previous nonattenders). All girls lived in Birmingham, United Kingdom, and (a) were registered with general practitioners within the BEN and Heart of Birmingham Primary Care Trusts; (b) were eligible to be vaccinated through the participating community clinics (Sutton Cottage, Partners in Health, and Dove Medical Centre); and (c) had not been vaccinated against HPV before.

### Recruitment and Randomization

**Figure 1 fig1:**
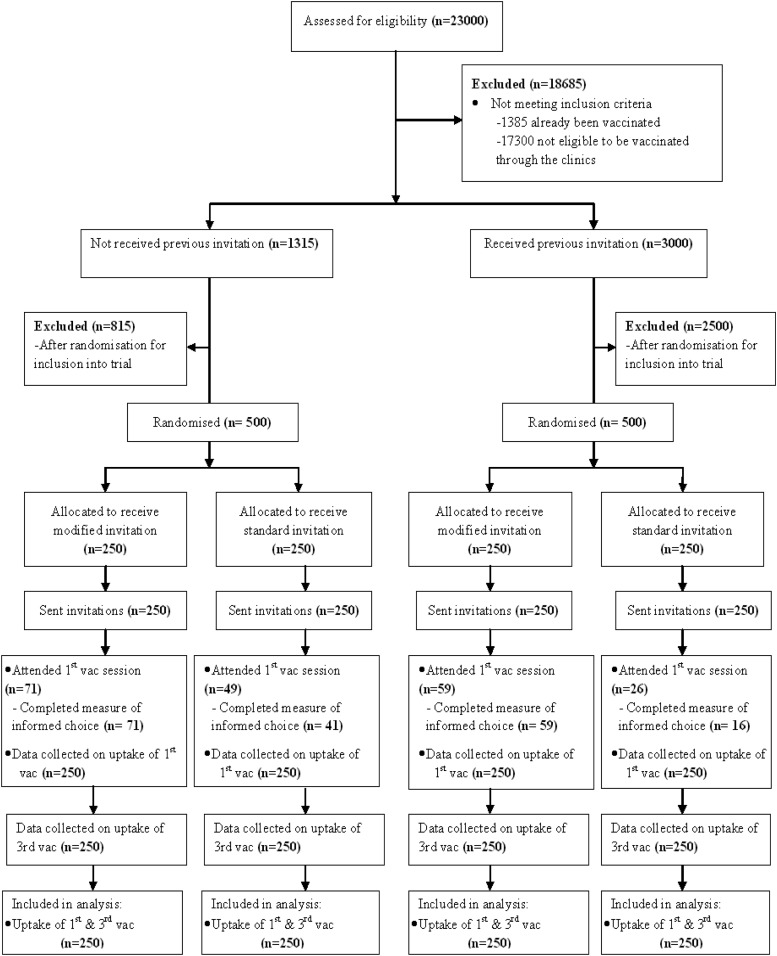
Consolidated Standards of Reporting Trials (CONSORT) flow diagram.

Participants were selected randomly from a list of names of all girls aged 16 to 18 years registered with participating general practitioners (see Figure 1). After excluding girls not meeting the inclusion criteria, the list was sorted according to whether girls were first-time invitees or previous nonattenders. Five hundred girls were randomly selected from each of these two sublists for inclusion in the trial, using the RAND() function in Excel, and were then randomized via the same technique to control versus intervention conditions (see Table 1).[Fig-anchor fig1][Table-anchor tbl1]

### Intervention

#### Invitation letters

All participants received letters, addressed to them, inviting them to attend their first HPV vaccination session. The letters included the date, time, and venue of their allocated vaccination appointment. Participants were given the option to reschedule their appointment or attend a different immunization clinic by contacting the immunization team at a designated telephone number, included in the letter.^3^

#### Information leaflet

Along with the invitation letters, all participants were sent a leaflet containing information about HPV and the HPV vaccine. This was the standard leaflet used and distributed by the NHS.^4^ It included information on the prevalence of HPV (i.e., that it is common, with most people getting infected at some point in their life), on how it spreads (i.e., through sexual activity with somebody who has the virus), on the different types of HPV that exist and their relationship to cervical cancer (i.e., that more than 100 types of HPV exist, but only 13 are known to cause cancer, with others being harmless or causing conditions such as genital warts), on the benefit of the HPV vaccine (i.e., that it reduces the risk of getting cervical cancer by 70%), on the limited protection afforded by it (i.e., that it protects against only the two types of the virus most often linked to cancer, but not against others or other sexually transmitted diseases, and does not prevent pregnancy), as well as on the consequences of getting vaccinated (i.e., the vaccine’s side effects—described as few and mild—and the continued need to undergo cervical cancer screening in the future). Participants wishing to obtain further information were directed to the relevant NHS Website.

#### Offer of financial incentives

Participants in the intervention groups received an invitation letter, which included the offer of Love2Shop vouchers worth £45 (€52; $65) for receiving the three vaccinations.^3^

The vouchers could be exchanged at numerous stores in the United Kingdom, including general merchandise and department stores; fashion and footwear retailers; specialist retailers (e.g., bookstores); jewelry shops; sports, outdoor, and motoring stores; home improvement and soft furnishing stores; restaurants; and leisure facilities (e.g., cinemas).^5^

The total amount was based on the only existing study of which we are aware, which assessed the impact of incentives on uptake of a vaccination requiring completion of three doses (Seal et al., 2003). In the Seal et al. study, participants were offered $60 (£40) for receiving three doses of the hepatitis B vaccine ($20 for each dose). Unlike this study, however, which offered fixed-value rewards for each vaccination, participants in the present study were offered larger rewards for receiving the first and third vaccination, in an attempt to motivate initiation and completion of the vaccination program. Specifically, they were offered £20 (€23; $29) for receiving the first vaccination, £5 (€6; $7) for the second vaccination, and £20 (€23; $29) for the third vaccination. The exact amounts offered for each vaccination were chosen through discussion with experts.

#### Reminder text messages

Participants in the intervention groups received text messages reminding them of their second and third vaccination sessions. These were sent during the intervals between the first and second vaccinations, and between the second and third vaccinations, and 2 days prior to the next session. The wording of these messages was, “(Name), don’t forget your HPV jab on (day) at (time) at the (venue). Thank you.” Participants were not able to reply to these messages. Participants in the control groups did not receive these reminder text messages.

### Outcome Measures

#### Uptake

Uptake of each vaccination was recorded at the community clinics where vaccinations took place and was measured as the proportion of those invited who received each vaccination.

#### Social deprivation

Area-level social deprivation was measured using participants’ postcodes to calculate English Index of Multiple Deprivation (IMD) scores, which range from 0.37 (least deprived) to 85.46 (most deprived) (Community & Neighborhoods, 2007). The IMD is a measure of deprivation in England based on area of residence. It measures deprivation at the small-area level, that is, the Lower Layer Super Output Area. It is derived from seven indices of deprivation, including income, employment, health deprivation and disability, education skills and training, barriers to housing, and services and crime. These are combined into a single deprivation score for each small area in England.

#### Quality of decisions to undergo vaccinations

To assess the impact of financial incentives on the quality of decisions to undertake the HPV vaccinations, a short modified version of a validated measure of informed choice was used (Marteau et al., 2001), consisting of the following:

1 Two items rated on a 7-point scale, assessing attitudes toward the HPV vaccination: “For me, having the HPV vaccination is (a) 1 (*not at all good*) to 7 (*extremely good*) and (b) 1 (*not at all harmful*) to 7 (*extremely harmful*).” Scores obtained on the latter item were reverse coded.
2 Three items assessing knowledge of the HPV vaccination. These items requested that participants determine the validity (whether “true” or “false”) of three statements relating to the vaccination: “If I have the HPV vaccination . . .,” (a) “I am less likely to get cervical cancer”; (b) “I am less likely to get other sexually transmitted diseases”; (c) “I am less likely to get pregnant.”

Participants were also requested to state their main reason for getting vaccinated.

The original measure of informed choice was developed for use in the context of prenatal screening and consists of eight items assessing knowledge and four items assessing attitudes (Marteau et al., 2001). The component scales have been shown to have good internal consistency, predictive validity, and discriminant validity (Michie et al., 2002). For the purposes of the present study, the knowledge items were adapted to assess awareness of the most important issues regarding uptake of the HPV vaccination, including the vaccine’s benefits and limited protection. These issues were highlighted in the information leaflet participants received. Their importance in comparison with the other information presented in the leaflet was judged and determined by a panel of experts. In order to reduce response burden, only three knowledge items were included (Cronbach’s α = .64). Similarly, only two out of the four original attitude items were chosen for inclusion—one assessing affective attitudes, requesting participants to indicate how good they think the HPV vaccination is and one assessing instrumental attitudes, requesting participants to indicate how harmful they think the HPV vaccination is. These were adapted from the original scale through rephrasing (Cronbach’s α = .63).

### Procedure

The financial incentive scheme was run by the BEN Primary Care Trust (in partnership with the Young Foundation). Participants were selected and recruited into the scheme by the Birmingham Primary Care Shared Services Agency, who holds and controls all patient data in Birmingham, United Kingdom. Recruitment took place between February and March of 2010.

The vaccination sessions were conducted at three community clinics (Sutton Cottage, Partners in Health, and Dove Medical Centre) between March and September 2010, by nurses working with Heart of Birmingham Primary Care Trust. When attending their first session, participants signed a consent form, completed the measure of informed choice, and selected a date for their next vaccination. Delivery of each vaccination was contingent on completion of all previous doses. After getting vaccinated, participants in the intervention groups were handed the appropriate vouchers.

### Research Governance

The intervention was run by Healthy Incentives, a social enterprise arising as a result of a partnership between the Young Foundation and the NHS BEN Primary Care Trust. The use of vaccination invitation letters, information leaflets, and reminder text messages were part of the latter’s standard health services. Also part of the Primary Care Trust’s standard practice is the use of records reflecting attendance in vaccination programs of all eligible patients registered within the Trust. These records are routinely used to create reports. The offer of financial incentives was introduced as part of the Primary Care Trust’s innovation in health service development and delivery. Permission to implement the incentive scheme was granted by the Primary Care Trust, which was responsible for all related insurance and indemnity arrangements. Participants’ anonymity and privacy were protected by the Data Protection Act (United Kingdom Parliament, 1998). The design that Healthy Incentives used was a Zelen design, which involves randomization before consent from participants has been sought. This is used particularly when evaluating the full, unbiased impact of screening interventions, in which knowledge of the trial by control groups may affect outcomes (Schellings et al., 2009; Torgerson & Roland, 1998; Zelen, 1979), such as in the present study. Ethical approval was sought for researchers at King’s College London to access data from the BEN Primary Care Trust in order to evaluate the financial incentives scheme and publish relevant findings. This was granted by the BEN Research Ethics Committee (reference 11/WM/0073, April 8, 2011). NHS Permission for Research was granted by the Birmingham and the Black Country Comprehensive Local Research Network (BBC CLRN) Research Management & Governance (RM&G) Consortium Office on behalf of the BBC CLRN RM&G Consortium Trusts (reference BENPC040.44791, August 1, 2011).

### Statistical Analysis

To assess the impact of the intervention on initial uptake (i.e., uptake of the first vaccination) and completion of the HPV-vaccination program (i.e., uptake of the third vaccination), logistic regressions were performed separately for first-time invitees and previous nonattenders. To test the moderating effect of social deprivation on the impact of the intervention, the interaction between IMD scores and intervention was added to the logistic regression models. To test whether there was a difference in the effect size of the intervention in the two samples, data sets were combined and another logistic regression was conducted, in which sample (i.e., first-time invitees vs. previous nonattenders) was added as a predictor of the model, along with the intervention. The chi-square test was used to test for differences in attrition rates from the first to the third vaccination between the intervention and control groups. Differences in knowledge of the HPV vaccination between intervention and control groups were tested using the chi-square test, and differences in attitudes toward the HPV vaccination were examined using one-way analysis of variance. All tests were assessed at the 5% level of significance.

## Results

All groups were comparable in age and social deprivation (see Table 2). Data met the linearity of the logit and multicollinearity assumptions required for the logistic regression analyses.[Table-anchor tbl2]

### Uptake of the First HPV Vaccination

Financial incentives significantly increased initial uptake of the HPV-vaccination program by approximately 10% (see Table 3) in both first-time invitees (*OR* = 1.63, 95% CI [1.075, 2.472]; see Table 4) and previous nonattenders (*OR* = 1.63, 95% CI [1.075, 2.472]; see Table 4). The effect size did not vary between the two samples (nonsignificant interaction between group [intervention vs. control] and previous invitation; *OR* = 0.611, 95% CI [0.319, 1.172], *p* > .05).[Table-anchor tbl3][Table-anchor tbl4]

### Uptake of the Third HPV Vaccination

The combination of financial incentives and text messages significantly increased completion of the HPV-vaccination program by about 10% (see Table 3) in both first-time invitees (*OR* = 2.152, 95% CI [1.324, 3.496]; see Table 4) and previous nonattenders (*OR* = 4.283, 95% CI [1.920, 9.551]; see Table 4). The size of effect was similar in the two samples (nonsignificant interaction between group and previous invitation; *OR* = 0.494, 95% CI [0.194, 1.257]; *p* > .05).

### Reduction in Uptake From First to Third Vaccination

**Figure 2 fig2:**
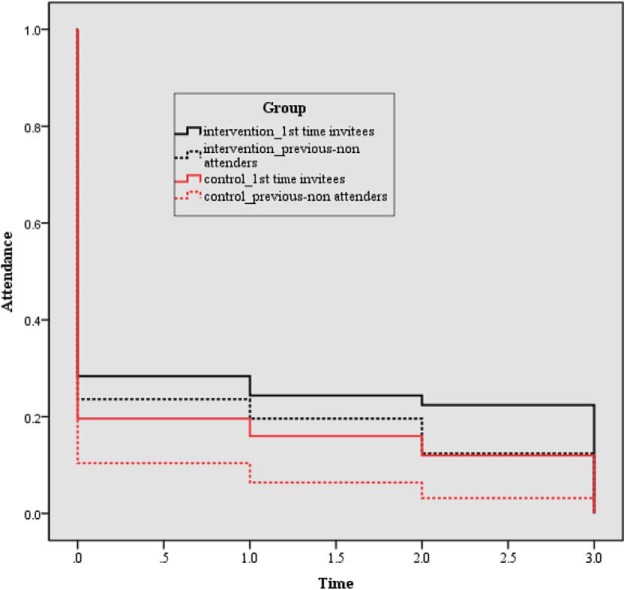
Drop-off in uptake of HPV vaccinations over time for each of the trial groups. The color version of this figure appears in the online article only.

Attrition between trial arms was similar. For first-time invitees, the attrition rate was 6% in the intervention group versus 7.6% in the control group, χ^2^(1, *n* = 500) = 0.50, *p* > .05. For previous nonattenders, the attrition rate was 11.2% in the intervention groups versus 7.4% in the control group, χ^2^(1, *n* = 500) = 2.39, *p* > .05 (see Figure 2).[Fig-anchor fig2]

### Social Deprivation

The effect of the intervention on uptake of the first and third vaccinations was not moderated by social deprivation in either first-time invitees (first vaccination: *OR* = 0.985, 95% CI [0.954, 1.017]; third vaccination: *OR* = 1.002, 95% CI [0.967, 1.038]) or previous nonattenders (first vaccination: *OR* = 0.998, 95% CI [0.76, 1.021]; third vaccination: *OR* = 1.007, 95% CI [0.966, 1.049]).

Social deprivation was unrelated to uptake of the first and third HPV vaccinations among first-time invitees. For previous nonattenders, higher levels of social deprivation were associated with a reduced chance of uptake for the third vaccination (*OR* = 0.980, 95% CI [0.964, 0.996], but not the first vaccination; see Table 4).

### Quality of Decisions

The quality of decisions to undergo the HPV vaccination was similar in the two trial arms: attitudes were similarly positive (first-time invitees: intervention group: *M* = 5.8, *SD* = 1.1, control group: *M* = 5.3, *SD* = 1.2; previous nonattenders: intervention group: *M* = 5.7, *SD* = 1.2, control group: *M* = 6.1, *SD* = 0.7, *F*[3, 188] = 1.203, *p* > .05.) and knowledge similarly high (first-time invitees: intervention group: 81.5% correct answers, control group: 84.5% correct answers; previous nonattenders: intervention group: 88.2% correct answers, control group: 87.5% correct answers), χ^2^ = (9, *n* = 193) = 9.017, *p* > .05.

Responses to a question about possible reasons for getting vaccinated also revealed no differences between groups (data available from authors).

## Discussion

Consistent with the hypotheses, the offer of financial incentives increased the proportion of girls undergoing an initial HPV vaccination, and the combination of financial incentives and reminder text messages increased the proportion of girls completing the course of three HPV vaccinations. Contrary to predictions, these effects did not vary with level of social deprivation. Also contrary to predictions, there was no evidence to suggest that girls’ attitudes toward the HPV vaccination and their knowledge of its health consequences were affected by the offer of financial incentives.

### Meaning of the Results

Findings from the current study are consistent with previous research demonstrating the effectiveness of financial incentives in promoting immunizations (Achat et al., 1999; Briss et al., 2000; Seal et al., 2003). Incentives may have operated by increasing the anticipated benefits of attending vaccination appointments sufficiently to overcome any perceived barriers. One of the barriers most strongly predicting uptake of the HPV vaccination is cost (Conroy et al., 2009). Although HPV vaccinations are free as part of normal care in the United Kingdom, getting vaccinated entails expenses, such as transport costs for attending clinics. Incentives may have operated by removing such financial barriers. Indeed, it has been shown that patients often use their rewards to cover expenses related to engaging in the target health behavior (Post, Cruz, & Harman, 2006). Girls in the current study could not use their incentives to directly pay for their transportation costs, as the incentives were offered in the form of vouchers. However, anecdotal evidence from a voucher-based incentive scheme targeting smoking cessation during pregnancy (Mantzari, Vogt, & Marteau, 2012a) suggests that incentivized individuals often engage in mental accounting, according to which transportation costs are deducted from the value of the vouchers in order to determine net gains.

The incentives increased uptake of the vaccination by 10%, a surplus that the intervention maintained throughout completion of the program, without reducing attrition between the first and third vaccinations. This finding could be taken as an indication of the superior effectiveness of the initial incentive, which may have been sufficient in maintaining the higher uptake rates without the subsequent incentives. It is not possible, however, to infer exactly how removal of the additional incentives would have affected the results. The relative effectiveness of the incentives for each HPV vaccination should be examined in future research.

One potential advantage of financial incentives is that they may be more effective in motivating behavior change in the most socially deprived (Sutherland et al., 2008). Contrary to such predictions, however, social deprivation did not moderate the effect of incentives in the present study. Perhaps the role of social deprivation depends on the type of behavior being targeted, and might therefore be limited in modifying the impact of incentives on uptake of the HPV vaccinations. It is also possible, however, that this finding is related to the measure of social deprivation used in this study. Although IMD scores have been previously used to assess participants’ level of deprivation (Marteau et al., 2010), current findings may have resulted from the use of a proxy rather than a direct measure of social deprivation. Alternatively, it is possible that the range of social deprivation in the current study was too limited to allow for an effect to be detected. Future studies should aim to include participants to reflect a wider range of socioeconomic statuses.

Although the intervention in the present study was effective in promoting the HPV vaccination, the highest uptake rates observed were 28.4% for the first vaccination and 22.4% for the third (by first-time invitees in the intervention group). These figures are well below the 80% uptake target set by the NHS. They are also considerably lower than the attendance rates for the catch-up program in the BEN Primary Care Trust, where this trial was run (72.2% for the first vaccination, and 34.2% for the third; Sheridan et al., 2010), as reported by the U.K. Department of Health (DH). The reasons underlying these differences in rates of uptake are unknown. A number of explanations are possible: First, they may reflect cohort differences. For example, the DH report refers to the attendance rates of 17- and 18-year-olds, whereas participants in the present study were aged 16 to 18 years (additional analyses revealed no effect of age on uptake). Second, they may reflect differences in the methods of patient recruitment and delivery of the HPV-vaccination programs. For example, delivery of HPV vaccinations through general practitioners is less effective compared with delivery through schools (Sheridan et al., 2010). Third, they may reflect issues with the reliability of the data reported in the DH report or the Primary Care Trust’s records. Anecdotal reports suggest that the latter’s records were not up-to-date, with girls who had already received the HPV vaccination through schools being invited for the present scheme; similar errors might have occurred when feeding figures into the DH report.

### Strengths

The main strength of this study is its novelty. It is the first study to assess the effectiveness of financial incentives for increasing uptake of the HPV vaccinations. It is also the first study, to our knowledge, that assesses deprivation level as a moderator of a financial incentive scheme. The incentive scheme used was designed to maximize retention: Unlike other studies that have offered fixed-value rewards for each vaccination (Seal et al., 2003), larger incentives (£20) were offered at the beginning and end of the program, to motivate participants to initiate and complete the vaccinations.

### Limitations

Although the results of this study demonstrate the effectiveness of financial incentives in increasing initial uptake of the HPV vaccination, they do not allow conclusive inferences to be made regarding the absolute impact of incentives on completion of the program. Girls in the intervention groups received reminder text messages prior to their second and third vaccination appointments. Due to an error made by the administration team, these reminder messages were not delivered to girls in the control groups. Vaccine recall and reminder systems are known to increase vaccination rates (Jacobson Vann & Szilagyi, 2005). It is therefore possible that the higher uptake rates of the third vaccination by the intervention groups are attributable to the reminder text messages, with incentives having no additional effect. This seems unlikely, given that incentives work best in combination with reminder systems and standing orders (Sutherland et al., 2008). Future research should examine the contribution of these interventions separately.

Our findings provide no evidence to suggest that financial incentives compromise the quality of decisions to engage in an incentivized behavior, with incentivized and nonincentivized girls’ attitudes toward the vaccination being similarly positive and their knowledge of its consequences similarly high. Perhaps the total amount girls could receive (£45) was not large enough to unduly influence their decision to get vaccinated had they held strong views against doing so. However, some limitations associated with the measurement of informed choice in this study reduce the certainty of the conclusions that can be drawn. First, as mentioned previously, we used a simplified version of a measure of informed choice originally developed and validated for use in the context of screening. The resulting measure may not have been sensitive enough to adequately assess informed choice in relation to HPV vaccination uptake. Further research is needed to validate the measure for use in this specific context. Second, the reliability of the measure was relatively low (Cronbach’s α = 0.63). Although related concerns might be partially alleviated by the questionable stability and appropriateness of the alpha coefficient for two-item scales (Cramer, Atwood, & Stoner, 2006; O’Brien, Buikstra, & Hegney, 2008; Sainfort & Booske, 2000; Verhoef, 2003), interpretation of the relevant findings nonetheless requires caution. Third, the measure relied on assessment of attitudes as a proxy of values, which, according to the operational definition of informed choice, need to be congruent with choices (Marteau et al., 2001). Although attitudes have been argued to reflect values (Marteau et al., 2001; Rokeach, 1968), the extent to which their measurement captures core values as traditionally conceptualized and measured (e.g., Rokeach, 1973; Schwartz, 1992) is unknown (Marteau, 2009). Furthermore, the measurement of attitudes alone provides little understanding of the potentially conflicting values individuals may hold when considering health-related decisions and how these affect their ability to make informed choices (Marteau, 2009). Further research is needed that will lead to the development of measures that better capture the values that underlie human health-related decisions (Marteau, 2009), which will, in turn, lead to more valid assessments of decision quality. Fourth, girls’ knowledge of the vaccination’s side effects was not assessed in this study. Consequently, no inferences can be made about whether the offer of a financial reward results in the risks associated with the incentivized behavior being overlooked (Marteau et al., 2009). Another limitation is related to the timing of assessment. Girls were requested to complete the measure of informed choice once they had decided to get vaccinated. It is therefore possible that their attitudes toward the vaccination might have been influenced by their decision to receive it, rather than being a predictor of that decision. Furthermore, assessing related attitudes and knowledge after the offer of financial incentives does not allow inferences to be made regarding the mechanisms by which incentives might have influenced the decision-making process. For example, it remains uncertain whether incentives facilitated girls with positive attitudes toward the vaccination to act in line with their values or whether the incentives changed the girls’ attitudes to make them more positive. Furthermore, by not having measures of attitudes by nonattenders, the insights afforded by such a comparison with attenders were not possible. Future research should aim to assess individuals’ knowledge and values related to an incentivized behavior before incentives are offered. Including subsequent behavior in the assessment, as proposed by Marteau et al. (2001), will allow for a more valid assessment of informed choice and will help elucidate the mechanisms by which incentives influence health-related decisions. Finally, our findings do not allow firm conclusions to be drawn regarding the possibility of incentives to negatively influence autonomy and people’s ability to voluntarily make decisions, and thus coerce them, as the measure of informed choice did not directly tap into these concepts. Further research should aim to complement the assessment of informed choice with measures that allow for a more direct and precise assessment of whether incentives are coercive and undermine autonomous choices, such as the Decisional Conflict Scale (O’Connor, 1995).

### Implications

While the incentive scheme increased uptake of HPV vaccinations, it is unknown whether such effects can be achieved in more cost-effective and acceptable ways. Other HPV-vaccination programs, such as those rolled out in schools (Sheridan et al., 2010), have achieved higher uptake rates than those achieved in this trial, without the expense of vouchers. Before the use of incentives is considered for wider implementation, research is needed to determine the optimal incentive value and delivery schedule for achieving maximum vaccination rates, supplemented with a formal cost-effectiveness analysis. Such cost-effectiveness analyses will need to take into account the extent to which the use of incentives might result in unintended consequences. These include (a) the potentially adverse effect of incentives on intrinsic motivation (Deci, Koestner, & Ryan, 1999), which might reduce the likelihood of individuals engaging in future health-related behaviors without the offer of rewards, and (b) the possibility for the offer of incentives to result in people refusing to adopt the incentivized behavior due to arousal of suspicion (Frey & Jegen, 2001).

Even if cost effective, the use of incentives for increasing HPV vaccinations will depend on their acceptability to policymakers, health professionals, and the public. The use of financial incentives for health promotion attracts negative views (Promberger, Brown, Ashcroft, & Marteau, 2011), which, coupled with the controversy surrounding the HPV vaccine for condoning early sexual activity (Waller, Marlow, & Wardle, 2006), may render the use of financial incentives for increasing HPV vaccinations unacceptable, as evidenced from the media coverage of the present scheme. For example, the *Daily Mail* online wrote, “HMV Voucher Bribe for Teenage Girls to Have Cervical Jabs: Fury at ‘Promiscuity Scheme’ as NHS Faces Cuts” (Martin, 2010), and wideshut.co.uk reported, “Girls Bribed to Take Dangerous and Pointless HPV Vaccine (Balderson, 2010).^7^

## Conclusion

The combination of financial incentives and reminder text messages increased uptake and completion of HPV vaccinations. Even with this intervention, however, the vaccination rates were considerably lower than the national target of 80%. Further research could attempt to elucidate the conditions under which the impact of incentives could be improved. However, it seems unlikely that the use of incentives alone will be sufficient to achieve effective HPV vaccination coverage targets in the United Kingdom. This, in addition to the cost effectiveness and acceptability issues surrounding the use of incentives in this context, perhaps highlight the need to consider alternative ways of achieving such targets.

## Figures and Tables

**Table 1 tbl1:** Description of Trial Groups

	First-time invitees	Previous nonattenders
Control group	250 (sent standard invitation letters; no incentives)	250 (sent standard invitation letters; no incentives)
Intervention group	250 (sent modified invitation letters; incentives)	250 (sent modified invitation letters; incentives)

**Table 2 tbl2:** Demographic Characteristics of Study Participants (Mean [SD])

Characteristic	First-time invitees		Previous nonattenders	
	Intervention	Control	Intervention	Control
Age	17.9 (0.76)	18.0 (0.69)	17.8 (0.81)	18.0 (0.74)
Social deprivation (IMD)	46.3 (13.12)	45.3 (13.0)	35.3 (21.9)	36.2 (22.2)
*Note.* IMD = Index of Multiple Deprivation.				

**Table 3 tbl3:** Proportion (% [N]) of Individuals in Each Sample and Within Each Group Receiving the Vaccinations

	First-time invitees		Previous nonattenders	
	Intervention (*n* = 250)	Control (*n* = 250)	Intervention (*n* = 250)	Control (*n* = 250)
First vac	28.4 (*n* = 71)	19.6 (*n* = 49)	23.6 (*n* = 59)	10.4 (*n* = 26)
Second vac	24.4 (*n* = 61)	16.0 (*n* = 40)	19.6 (*n* = 49)	6.4 (*n* = 16)
Third vac	22.4 (*n* = 56)	12.0 (*n* = 30)	12.4 (*n* = 31)	3.0 (*n* = 8)
*Note.* vac = vaccination.				

**Table 4 tbl4:** OR and CIs of Group and IMD for First-Time Invitees and Previous Nonattenders for the First and Third Vaccinations

	First-time invitees				Previous nonattenders			
	*B* (*SE*)	*OR*	95% CI for *OR*		*B* (*SE*)	*OR*	95% CI for *OR*	
			Lower	Upper			Lower	Upper
First vac								
Group	0.489 (0.212)	1.630*	1.075	2.472	0.976 (0.255)	2.654*	1.609	4.378
IMD	−0.002 (0.008)	0.998	0.983	1.014	−0.004 (0.006)	0.996	0.985	1.007
Third vac								
Group	0.766 (0.248)	2.152*	1.324	3.496	1.455 (0.409)	4.283*	1.920	9.551
IMD	−0.013 (0.014)	0.987	0.970	1.004	−0.020 (0.008)	0.980*	0.964	0.996
*Note.* *OR* = odds ratio; CI = confidence interval; IMD = Index of Multiple Deprivation; vac = vaccination.								
* *p* < .05.								

## Footnotes

1 The National Health Service (NHS) is a publicly funded health care system within the United Kingdom, which provides a comprehensive range of health services, the vast majority of which are free at the point of use for residents of the United Kingdom of England, Scotland, Wales, and Northern Ireland. HPV vaccinations are provided free through the NHS to girls aged 12 to 18 years of age. The HPV vaccine is delivered largely through secondary schools but is also offered through general practitioners (primary care physicians) and community clinics. Parental consent is not required for vaccinating adolescents over 16 years of age.

2 Published protocol available at www.biomedcentral.com/1472-6963/12/301

3 An example of the letter is available at http://www.biomedcentral.com/content/supplementary/1472-6963-12-301-s1.pdf

4 An example of the leaflet is available at http://www.dhsspsni.gov.uk/17-18.pdf

5 For more information, visit http://www.highstreetvouchers.com/gift-vouchers/love2shop

7 These media reports were published in November 2010, after completion of the program, which ended in September 2010, limiting the possibility that they may have confounded the results of the study.
